# Berberine alleviates myocardial ischemia–reperfusion injury by inhibiting inflammatory response and oxidative stress: the key function of miR-26b-5p-mediated PTGS2/MAPK signal transduction

**DOI:** 10.1080/13880209.2022.2048029

**Published:** 2022-03-20

**Authors:** Xiaojing Jia, Wei Shao, Suqing Tian

**Affiliations:** a Department of Pharmacy, The First People’s Hospital of Lianyungang, Lianyungang, China; b Department of Pharmacy, Pingyi County Hospital of Traditional Chinese Medicine, Linyi, China; c Department of Pharmacy, Jining Hospital of Traditional Chinese Medicine, Jining, China

**Keywords:** Cardiovascular disease, inflammation, microRNA, natural compound

## Abstract

**Context:**

Berberine has myocardial protective effects.

**Objectives:**

The protective effects of berberine on heart ischemia–reperfusion (I/R) injury were explored.

**Materials and methods:**

Human cardiomyocytes were divided into control group, oxygen-glucose deprivation/re-oxygen (OGD/R) (2 h OGD with 24 h reoxygenation) group, OGD/R + low group (5 μM berberine for 24 h) and OGD/R + high group (10 μM berberine for 24 h). Twenty-four Wistar rats were divided into sham group, I/R group (45 min occlusion with 2 h reperfusion), I/R + berberine group (50 mg/kg berberine 1 h before I/R surgery) and I/R + berberine + antagomir (intraperitoneally injected with miR-26b-5p antagomir). MicroRNA profile, effects of berberine on I/R or OGD/R-induced injuries, and the role of miR-26b-5p in the function of berberine were explored.

**Results:**

OGD/R treatment suppressed viability (0.41 ± 0.05 vs. 0.87 ± 0.13, *p*< 0.05), while induced apoptosis (6.6 ± 1.0% vs. 26.3 ± 4.8%, *p*< 0.05) in cardiomyocytes, which was restored by berberine (viability: 0.64 ± 0.01 for 5 μM and 0.72 ± 0.01 for 10 μM, *p*< 0.05; apoptosis: 10.9 ± 2.2 for 5 μM and 7.9 ± 1.3 for 10 μM). Berberine induced miR-26b-5p and inhibited PTGS2/MAPK pathway. MiR-26b-5p inhibition counteracted the protective function of berberine. In rats, berberine (50 mg/kg) improved heart histological structure and suppressed inflammatory response, which was impaired by miR-26b-5p inhibition.

**Discussion and conclusions:**

Berberine exerted anti-I/R function in heart by inducing miR-26b-5p and suppressing the PTGS2/MAPK pathway. These data promote the application of berberine as an anti-I/R agent.

## Introduction

Acute myocardial injury (AMI) is a major causable factor contributing to disease-related death worldwide (Keeley et al. 2003). The attack of AMI results in a deficient supply of cardiac blood, which is associated with the lack of oxygen and nutrients in myocardial tissues. Thus, the re-establishment of the blood supply is a central issue in the clinical management of AMI. Currently, the most widely employed strategies for handling AMI are those based on the application of primary percutaneous coronary intervention (PPCI) and thrombolytic therapy. However, these treatments can themselves lead to secondary injury in myocardial tissue, which is recognized as ischemia–reperfusion (I/R) injury (IRI) (Peuhkurinen 1989; Piper 1998; Yellon and Hausenloy 2007). Unlike the treatment for AMI, effective handling approaches for IRI are lacking in the clinic. In this respect, IRI remains a neglected therapeutic target for cardioprotection of patients treated with PPCI, and the development of effective therapies for attenuating impairments associated with IRI is in great demand.

Phytochemicals have shown promising potential in improving cardiac status, and there has been increasing interest in assessing the effect of different plant-derived compounds in protecting myocardial tissues. Of numerous compounds, berberine is a nonbasic, quaternary benzylisoquinoline plant alkaloid and has been applied in Ayurvedic and Chinese medicinal systems for centuries. The compound can be isolated from several herbs, such as *Hydrastis canadensis* Linn. (Ranunculaceae), *Cortex phellodendron* Schneid (Rutaceae) and *Coptis chinensis* Franch (Ranunculaceae) (Inoki et al. 2003), and has shown considerable function in modulating oxidative stress, immune response and neurotransmitters and in antagonizing cancers (Kumar et al. 2015). Regarding its effect on myocardial tissue, previous studies have revealed its protective effect on myocardial tissues from multipronged aspects. For instance, it was shown that berberine could reduce IRI-induced myocardial apoptosis by activating AMPK and PI3K–Akt signalling (Chen et al. 2014). Another study showed that berberine could alleviate cardiac IRI by inhibiting excessive autophagy (Huang et al. 2015). The myocardial protective effect has even been verified by some clinical investigations: a 2 month treatment with berberine could evidently improve the cardiac function of antiarrhythmic patients (Zeng et al. 2003). Collectively, berberine has shown promising potential in protecting hearts against different impairments, including IRI. Thus, a more comprehensive understanding of the mechanism underlying the anti-IRI effect of berberine will facilitate its application in practice.

Accumulating evidence shows that the chronic inflammatory response is an important promoting factor in cardiovascular diseases (Smeets et al. 2008). This notion has been verified with myocardial symptoms such as hypertrophy and fibrosis (Nicoletti and Michel 1999; Ren et al. 2007; Rorabaugh et al. 2020). Regarding the attack of IRI, injuries will induce inflammation, and inflammation will strengthen the injury-induced impairments in return (Yu et al. 2017; Al-Salam and Hashmi 2018). Therefore, the suppression of inflammation associated with IRI may contribute to the recovery of cardiac function and structure.

MicroRNAs (miRs) are a class of noncoding RNAs and are involved in the modulation of multiple biological processes (Bang et al. 2014), including the inflammatory response and oxidative stress. However, the role of miRs in the anti-inflammatory and cardioprotective functions of berberine has barely been explored. Thus, it is reasonable to assess the interaction between berberine and miRs in an IRI system, which will not only facilitate the application of berberine but also provide novel therapeutic targets for the attenuation of IRI. In the current study, we performed a microarray assay to determine miRs responding to berberine treatment in IRI cardiomyocytes and selected the one with the highest sensitivity, which is miR-26b-5p. Then, the role of miR-26b-5p-mediated PTGS2/MAPK pathway (Ge et al. 2019) in the protective function of berberine against myocardial IRI was determined with a series of *in vitro* and *in vivo* assays.

## Materials and methods

### Chemicals and agents

Berberine was purchased from MedChemExpress (Monmouth Junction, NJ) and dissolved in saline. Agents for PCR detection, including RNA Purified Total RNA Extraction Kit (RP1201) and super M-MLV reverse transcriptase (RP6502) were purchased from BioTeke (Beijing, China). Agents for western blotting detection, including PMSF lysis (P0013B), protein concentration determination kit (P0012), cell cycle detection kit (C1052), CCK-8 detection kit (C0037) and Hoechst staining kit (C0003) were purchased from Beyotime Biotechnology (Shanghai, China). Cell apoptosis detection kit (KGA106) was purchased from KeyGen BioTECH (Nanjing, China). Detection kits for malondialdehyde (MDA) (A003-1), glutathione (GSH) (A006-2), glutathione peroxidase (GSH-Px) (A005) and superoxide dismutase (SOD) (A001-1) were obtained from Nanjing Jiancheng Bioengineering Institute (Nanjing, China). Detection kits for tumour necrosis factor-α (TNF-α) (EK3821/2), interleukin-1β (IL-1β) (EK301B1/2) and IL-6 (70-EK206) were purchased from Multi Sciences (Hangzhou, China).

### Cell culture and OGD/R treatment

Human cardiomyocytes were purchased from American Type Culture Collection (ATCC) (Rockville, MD) and cultured in DMEM/F-12 medium supplemented with 10% (v/v) foetal calf serum (FCS) in an atmosphere consisting of 95% air and 5% CO_2_ at 37 °C. I/R injures was imitated by subjecting cardiomyocytes to oxygen-glucose deprivation/re-oxygen (OGD/R) administration: briefly, log-growth cardiomyocytes were adjusted to a concentration of 5 × 10^4^/mL and incubated on slides in one-well of 24-well plates for 24 h. Afterwards, cells were subjected to OGD treatment for two hours before incubated with medium containing normal oxygen (21%) for 24 h for reoxygenation.

### Microarray detection and reverse transcription quantitative PCR (RT-qPCR)

To determine the potential miR target for berberine treatment, total RNA in OGD/R-treated cardiomyocytes and cardiomyocytes co-treated OGD/R and berberine of 10 μM was extracted using TRIzol method (Gibco/BRL, Carlsbad, CA). On the microfluidic chip, each detection probe consisted of a chemically modified nucleotide coding segment complementary to target miR (from miRBase, http://microrna.sanger.ac.uk/sequences) or other RNA (control or customer defined sequences) and a spacer segment of polyethylene glycol to extend the coding segment away from the substrate. The data output was received in Excel spreadsheets containing the normalized miR expression profiles. Differentially expressed miRs in berberine-treated injured cardiomyocytes were filtered to exclude those changing less than 2.0-fold compared with those in injured cardiomyocytes (Table 1).

**Table 1.** Raw data of microarray detection.

Systematic name	*p* Value	Fold change	Regulation	active_sequence	mirbase_accession_No
hsa-miR-196a-5p	1.36167E–07	0.029277281	Down	CCCAACAACATGAAACTACC	MIMAT0000226
hsa-miR-654-3p	3.57491E–07	3.144111196	Up	AAGGTGATGGTCAGCAGAC	MIMAT0004814
hsa-miR-126-3p	1.13657E–05	0.300904176	Down	CGCATTATTACTCACGGT	MIMAT0000445
hsa-miR-26b-5p	0.009093291	12.47704786	Up	ACCTATCCTGAATTACTTGA	MIMAT0000083
hsa-miR-10a-5p	1.64084E–05	0.09945425	Down	CACAAATTCGGATCTACAGGG	MIMAT0000253
hsa-miR-4306	4.27277E–05	0.370964359	Down	TACTGCCTTTCTCTCCA	MIMAT0016858
hsa-miR-27b-3p	6.29176E–05	0.215713452	Down	GCAGAACTTAGCCACTGT	MIMAT0000419
hsa-miR-29a-3p	7.01052E–05	14.39136436	Up	TAACCGATTTCAGATGGTGC	MIMAT0000086
hsa-miR-195-5p	7.71526E–05	0.362171203	Down	GCCAATATTTCTGTGCTGC	MIMAT0000461
hsa-miR-1268a	8.49446E–05	0.46110227	Down	CCCCCACCACCAC	MIMAT0005922
hsa-miR-199a-3p	9.50825E–05	29.33406516	Up	TAACCAATGTGCAGACTACT	MIMAT0000232
hsa-miR-29b-3p	0.000134054	14.68487296	Up	AACACTGATTTCAAATGGTGC	MIMAT0000100
hsa-miR-424-5p	0.000178202	8.100486982	Up	TTCAAAACATGAATTGCTGCTG	MIMAT0001341
hsa-miR-199a-5p	0.000194277	3.58342415	Up	GAACAGGTAGTCTGAACAC	MIMAT0000231
hsa-miR-155-5p	0.00021575	11.58014237	Up	ACCCCTATCACGATTAG	MIMAT0000646
hsa-miR-224-3p	0.000264127	0.33997565	Down	TGTAGTCACTAGGGCAC	MIMAT0009198
hsa-miR-31-5p	0.000277092	2.844173972	Up	AGCTATGCCAGCATCTT	MIMAT0000089
hsa-miR-221-3p	0.00030458	6.191406188	Up	GAAACCCAGCAGACAATGT	MIMAT0000278
hsa-miR-210-3p	0.00036914	0.228172338	Down	TCAGCCGCTGTCACAC	MIMAT0000267
hsa-miR-31-3p	0.000399566	2.582710822	Up	ATGGCAATATGTTGGCATAG	MIMAT0004504
hsa-miR-196b-5p	0.00046877	2.261225558	Up	CCCAACAACAGGAAACTACC	MIMAT0001080
hsa-miR-376a-3p	0.00067719	15.70451694	Up	ACGTGGATTTTCCTCTATG	MIMAT0000729
hsa-miR-29b-1-5p	0.000707109	4.093897383	Up	TCTAAACCACCATATGAAACCAG	MIMAT0004514
hsa-miR-181b-5p	0.000730886	0.365436516	Down	ACCCACCGACAGCA	MIMAT0000257
hsa-miR-376c-3p	0.000765382	17.2507126	Up	ACGTGGAATTTCCTCTATG	MIMAT0000720
hsa-miR-361-5p	0.000955274	3.177196425	Up	GTACCCCTGGAGATTC	MIMAT0000703
hsa-miR-4788	0.000972908	0.464275958	Down	GCCTCCCTTAGCTGG	MIMAT0019958
hsa-miR-23b-3p	0.0010275	0.135112416	Down	GGTAATCCCTGGCAATG	MIMAT0000418
hsa-miR-331-3p	0.001062311	0.185272503	Down	TTCTAGGATAGGCCCAGGG	MIMAT0000760
hsa-miR-140-3p	0.001232036	2.086498641	Up	CCGTGGTTCTACCCT	MIMAT0004597
hsa-miR-409-3p	0.001242163	2.324297722	Up	AGGGGTTCACCGAGCA	MIMAT0001639
hsa-miR-18a-5p	0.001456004	2.333173168	Up	CTATCTGCACTAGATGCA	MIMAT0000072
hsa-miR-222-3p	0.001618366	3.316027749	Up	ACCCAGTAGCCAG	MIMAT0000279
hsa-miR-96-5p	0.001675276	0.160294429	Down	AGCAAAAATGTGCTAGTGCCAA	MIMAT0000095
hsa-miR-299-5p	0.001677551	3.321531812	Up	ATGTATGTGGGACGGTAAAC	MIMAT0002890
hsa-miR-381-3p	0.001808418	3.151358113	Up	ACAGAGAGCTTGCCCT	MIMAT0000736
hsa-miR-148a-3p	0.002098112	0.106429762	Down	ACAAAGTTCTGTAGTGCACT	MIMAT0000243
hsa-miR-6749-5p	0.002348973	2.590749142	Up	GCTCCCCCAACCC	MIMAT0027398
hsa-miR-1273g-3p	0.002389208	2.094061546	Up	CTCAGGCTGGAGTGC	MIMAT0022742
hsa-miR-140-5p	0.002573786	3.440831337	Up	CTACCATAGGGTAAAACCACT	MIMAT0000431
hsa-miR-379-5p	0.002755251	3.636451885	Up	CCTACGTTCCATAGTC	MIMAT0000733
hsa-miR-181a-5p	0.002769545	0.177984883	Down	ACTCACCGACAGCGT	MIMAT0000256
hsa-miR-98-5p	0.002777891	0.286369402	Down	AACAATACAACTTACTACCTC	MIMAT0000096
hsa-miR-10b-5p	0.002938805	4.380494946	Up	CACAAATTCGGTTCTACAGGG	MIMAT0000254
hsa-miR-224-5p	0.002948588	0.103337649	Down	AACGGAACCACTAGTGACTT	MIMAT0000281
hsa-miR-7-5p	0.003301142	2.07913788	Up	ACAACAAAATCACTAGTCTTCC	MIMAT0000252
hsa-miR-183-5p	0.003424888	0.24131117	Down	AGTGAATTCTACCAGTGCCA	MIMAT0000261
hsa-miR-425-5p	0.003699655	0.383324012	Down	TCAACGGGAGTGATCGTG	MIMAT0003393
hsa-miR-218-5p	0.003701553	9.470921863	Up	ACATGGTTAGATCAAGCACA	MIMAT0000275
hsa-miR-335-5p	0.004020134	3.20834286	Up	ACATTTTTCGTTATTGCTC	MIMAT0000765
hsa-miR-452-5p	0.004096302	0.207007477	Down	TCAGTTTCCTCTGCAAA	MIMAT0001635
hsa-miR-137	0.004363633	3.507386479	Up	CTACGCGTATTCTTAAGCAA	MIMAT0000429
hsa-miR-125b-5p	0.005370642	4.027400002	Up	TCACAAGTTAGGGTCTC	MIMAT0000423
hsa-miR-582-5p	0.005532631	0.183941311	Down	AGTAACTGGTTGAACAACTGTA	MIMAT0003247
hsa-miR-193b-3p	0.005977816	0.301279354	Down	AGCGGGACTTTGAGGG	MIMAT0002819
hsa-miR-100-5p	0.00604851	2.188791677	Up	CACAAGTTCGGATCTACGG	MIMAT0000098
hsa-let-7d-5p	0.006239721	0.241051824	Down	AACTATGCAACCTACTACC	MIMAT0000065
hsa-miR-154-3p	0.006273607	3.30210135	Up	AATAGGTCAACCGTGTATGA	MIMAT0000453
hsa-let-7b-5p	0.00645594	0.328648059	Down	AACCACACAACCTACTACC	MIMAT0000063
hsa-miR-22-3p	0.006467066	2.317910024	Up	ACAGTTCTTCAACTGGCAG	MIMAT0000077
hsa-miR-19a-3p	0.00711885	2.89409166	Up	TCAGTTTTGCATAGATTTGCA	MIMAT0000073
hsa-miR-221-5p	0.007292288	3.973215915	Up	AAATCTACATTGTATGCCAGG	MIMAT0004568
hsa-miR-365a-3p	0.008659997	0.290248955	Down	ATAAGGATTTTTAGGGGCATTA	MIMAT0000710
hsa-miR-34b-5p	0.009411234	3.985790347	Up	CAATCAGCTAATGACACTGCCT	MIMAT0000685
hsa-miR-5100	0.009800681	7.722254528	Up	AGAGGCACCGCTGG	MIMAT0022259
hsa-miR-4687-3p	0.00994601	2.186452921	Up	GCCTGCCCCCTCC	MIMAT0019775
hsa-miR-487b-3p	0.010401911	2.217521262	Up	AAGTGGATGACCCTGTAC	MIMAT0003180
hsa-miR-758-3p	0.010411478	2.972554804	Up	GGTTAGTGGACCAGGTCAC	MIMAT0003879
hsa-miR-377-3p	0.011993679	3.358651972	Up	ACAAAAGTTGCCTTTGTGTG	MIMAT0000730
hsa-miR-6869-5p	0.013118543	2.038982087	Up	GCCGCCGCGC	MIMAT0027638
hsa-miR-1587	0.016447548	0.452334043	Down	CCCAACCCAGCCC	MIMAT0019077
hsa-miR-4455	0.020420972	0.428379795	Down	AAAAACACACACACCCT	MIMAT0018977
hsa-miR-4286	0.025385424	3.090528337	Up	GGTACCAGGAGTGGG	MIMAT0016916
hsa-miR-6821-5p	0.026376124	0.187451796	Down	CCCCGCCTCGAG	MIMAT0027542
hsa-let-7f-5p	0.027525902	0.422518245	Down	AACTATACAATCTACTACCTC	MIMAT0000067
hsa-let-7c-5p	0.029515864	0.498725999	Down	AACCATACAACCTACTACC	MIMAT0000064
hsa-miR-7977	0.033035454	2.017042364	Up	TGGTGCGTTGGCTG	MIMAT0031180
hsa-miR-4507	0.039495482	0.344331612	Down	CCCAGCCCAGCC	MIMAT0019044

RNA was extracted using an extraction kit according to the manufacturer’s instruction and cDNA templates were achieved using super M-MLV reverse. The reaction mixture of qPCR contained 10 μL SYBR GREEN mastermix, 0.5 μL of each primer (Table 2), 1 μL cDNA template and 8 μL ddH_2_O. Amplification was performed in Exicycler™ 96 (BIONEER, Daejeon, South Korea) using following parameters: a denaturation step at 94 °C for 10 min, followed by 40 cycles of amplification at 94 °C for 10 s, 60 °C for 20 s and 72 °C for 30 s, then the reaction was stopped at 25 °C for 1 min. The signal was detected after the step at 72 °C for 30 s. The relative expression level of miR-26b-5p was calculated according to the formula of 2^–ΔΔCt^ in reference to control group.

**Table 2.** Primer information.

Gene	Direction	Sequence (5′–3′)
miR-26b-5p	Forward	TATCTAGACATCTGCTACCTCCTCCC
	Reverse	ATGCGGCCGCGATTCAACAAGGACAA
U6	Forward	CTCGCTTCGGCAGCACA
	Reverse	AACGCTTCACGAATTTGCGT

### Cell grouping

To assess the protective effect of berberine against OGD/R administration, cardiomyocytes were grouped as following: control group, normal cardiomyocytes; OGD/R group, cells underwent OGD/R treatment; OGD/R + low group, cells were incubated with 5 μM berberine for 24 h before OGD/R treatment (Table 3); OGD/R + high group, cells were incubated with 10 μM berberine for 24 h before OGD/R treatment (Table 3). Additionally, the role of miR-26b-5p in the protective effect of berberine was further assessed by inhibiting the expression level of the miR with specific inhibitor (5′-UUGUUCAUAUAGAUCUCGUCAUU-3′) (inhibitor oligonucleotides purchased from Sangon Biotech, Shanghai, China) in cardiomyocytes before OGD/R and berberine treatment of 10 μM.

**Table 3.** Determination of treating concentration of berberine *in vitro* assays.

Time	Concentration							
	0 μM		5 μM		10 μM		20 μM	
12 h	0.31	0.05	0.31	0.06	0.44	0.09	0.46	0.09
24 h	0.37	0.06	0.58	0.11	0.74	0.12	0.75	0.11
36 h	0.44	0.09	0.63	0.09	0.79	0.11	0.84	0.10
48 h	0.49	0.10	0.69	0.11	0.82	0.12	0.88	0.09

### CCK-8 assay

After different treatments, cell viability was determined using CCK-8 assay: exponentially growing cardiomyocytes were incubated with CCK-8 solution (10 μL) at 37 °C for 1 h. The OD values at 450 nm (OD_450_) were detected with a Microplate Reader (ELX-800, BIOTEK, Winooski, VT) and employed as the representative of cell viability.

### Cell apoptosis detection

The apoptotic rate was determined using a cell apoptosis detection kit with a FACScan flow cytometer (Accuri C6, BD, Franklin Lakes, NJ): briefly, cells were incubated with 5 μL Annexin V for 10 min and then incubated with 5 μL propidium iodide. The total apoptotic rate of the cell lines was equal to the sum of the cell death rate (UR, upper right quadrant) and the early apoptosis rate (LR, lower right quadrant).

### Inflammation and oxidative stress measurement

The production of MDA, GSH, GSH-Px, SOD, TNF-α and IL-1β in cardiomyocytes and in animal samples was measured using specific kits following the instructions of manufacturers.

### Western blotting assay

Total protein from human cardiomyocytes and rat heart tissues was collected by subjecting the samples to 1% PMSF and the concentrations of the protein samples were determined using BCA method. Protein (30 μg) from each sample was subjected to 10% sodium dodecyl sulphate polyacrylamide gel electrophoresis (SDS-PAGE) at 80 V for 2.5 h and then transferred onto polyvinylidene difluoride (PVDF) membranes. After being blocked with skimmed milk solution for 1 h, the membranes were first incubated with the primary antibodies (Table 4) at 4 °C overnight and then incubated with secondary antibodies (Table 4) for 45 min at 37 °C. The protein bands were developed using the Beyo ECL Plus reagent and the images were captured in the Gel Imaging System. The integrated optical density (IOD) of proteins was analysed by the Gel-Pro-Analyzer (Media Cybernetics, Rockville, MD) and the relative expression levels of proteins were calculated in reference to control group with GAPDH as the internal reference protein.

**Table 4.** Antibody information.

Protein	Dilution	Company, country
PTGS2	1:1000	Abcam, China
p-ERK1/2	1:2000	Abcam, China
ERK1/2	1:1000	Abcam, China
p-JNK	1:2000	Abcam, China
JNK	1:500	Abcam, China
p-p38	1:1000	Abcam, China
p38	1:1000	Abcam, China
Bax	1:1000	Boster, China
Bcl-2	1:1000	Boster, China
GAPDH	1:1000	Abcam, China
Secondary goat-anti rabbit antibody	1:5000	Beyotime Biotechnology, China
Secondary goat-anti mouse antibody	1:5000	Beyotime Biotechnology, China

### Myocardial I/R injured rat model establishment

Eight-week-old male Wistar rats (weighing 240–260 g) were maintained in cages at room temperature (20–25 °C) with free access to food and water. All animal experiments were conducted in accordance with the Institutional Animal Ethics Committee of Jining Hospital of Traditional Chinese Medicine (approval no. 20190023) and Animal Care Guidelines for the Care and Use of Laboratory Animals published by the US National Institutes of Health (NIH Publication No. 85-23, revised 1996). Twenty-four male rats were randomly divided into four groups (six for each group): sham group, rats underwent chest open but without anterior descending (LAD) artery ligation; I/R group, the LAD artery of rats was reversibly occluded for 45 min followed by 2 h of reperfusion; I/R + berberine group, rats were gavaged with 50 mg/kg body weight berberine 1 h before I/R surgery; I/R + berberine + antagomir group, rats were intraperitoneally injected with miR-26b-5p antagomir 24 h before berberine and I/R administrations.

### Detection of myocardial function and structure

The cardiac function of rats in different groups was determined by measuring haemodynamics parameters and myocardial levels of lactate dehydrogenase (LDH) and creatine kinase (CK). Upon completion of the experimental period, rats were anaesthetized with pentobarbital sodium (50 mg/kg). Then, the left ventricular end-systolic pressure (LVESP), left ventricular end-diastolic pressure (LVEDP), left ventricular end-systolic diameter (LVESD), the left ventricular end-diastolic dimension (LVEDD) and fractional shortening (FS) were measured. Moreover, the electrocardiogram (ECG) patterns were recorded by Animal ECG Acquisition and Analysis System SP2006 (Ruanlong Biotechnology Co., Ltd., Beijing City, China) in anaesthetized rats. Afterwards, rats were killed using overdose pentobarbital sodium and the myocardial levels of lactate (LDH) and CK were measured using LDH and CK assay kits (Nanjing Jiancheng Bioengineering Institute, Nanjing, China) according to the manufacturer’s instructions. Histological changes of ischaemic penumbra part of heart tissues were observed using H&E staining: briefly, samples were fixed in 10% neutral formalin and embedded in paraffin, and 5 μm sections were then stained with haematoxylin and eosin (H&E) (Solarbio, Beijing, China).The results were detected under microscope at ×200.

### Statistical analysis

All data were expressed in the form of mean ± standard deviation (SD). One-way analysis of variance (ANOVA) was performed, which was followed by multiple comparisons using Tukey’s method. All statistical analyses and graph manipulation were conducted using GraphPad Prism version 6.01 for windows (GraphPad Software, La Jolla, CA) with a significant level of 0.05.

## Results

### Expression profile of miRs and target gene prediction

**Figure 1.**
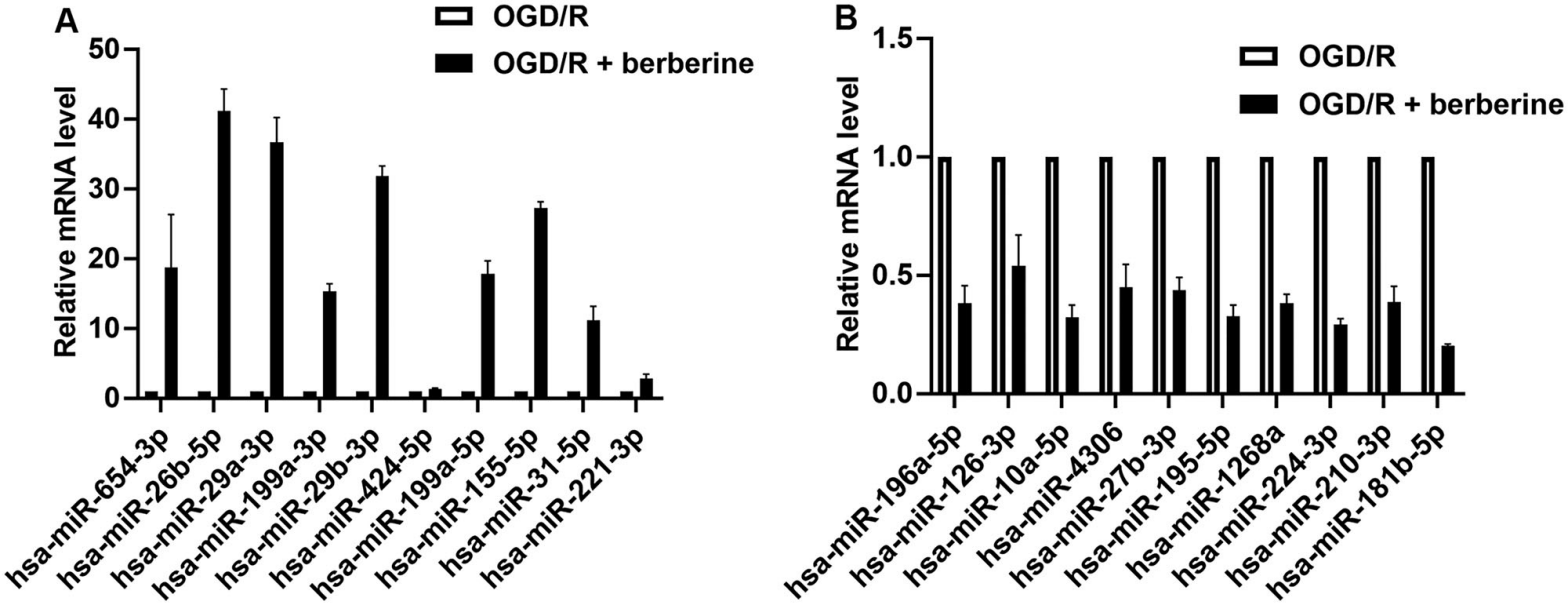
RT-qPCR validation of miR expression levels. The expression levels of 10 miRs in either upregulated members (A) or downregulated members (B) were validated by RT-qPCR.

Following the raw microarray data, 45 miRs were upregulated and 33 miRs were downregulated in cardiomyocytes cotreated with OGD/R and berberine compared with cardiomyocytes treated with OGD/R only (Table 1). To determine the potential target mediating the anti-I/R effect of berberine, the expression levels of 10 miRs in either upregulated members or downregulated members were validated by RT-qPCR (Figure 1). The potential target by berberine was selected based on the *p* value calculated using the relative expression level and target genes that were related to the progression of inflammation. Collectively, the current study selected miR-26b-5p as the potential target by berberine: the fold change of miR-26b-5p showed the lowest *p* value when compared with other miRs. Moreover, miR specifically regulates the expression of PTGS2, which is a well-characterized proinflammatory factor.

### Berberine increased viability while suppressing apoptosis in OGD/R-treated cardiomyocytes

**Figure 2.**
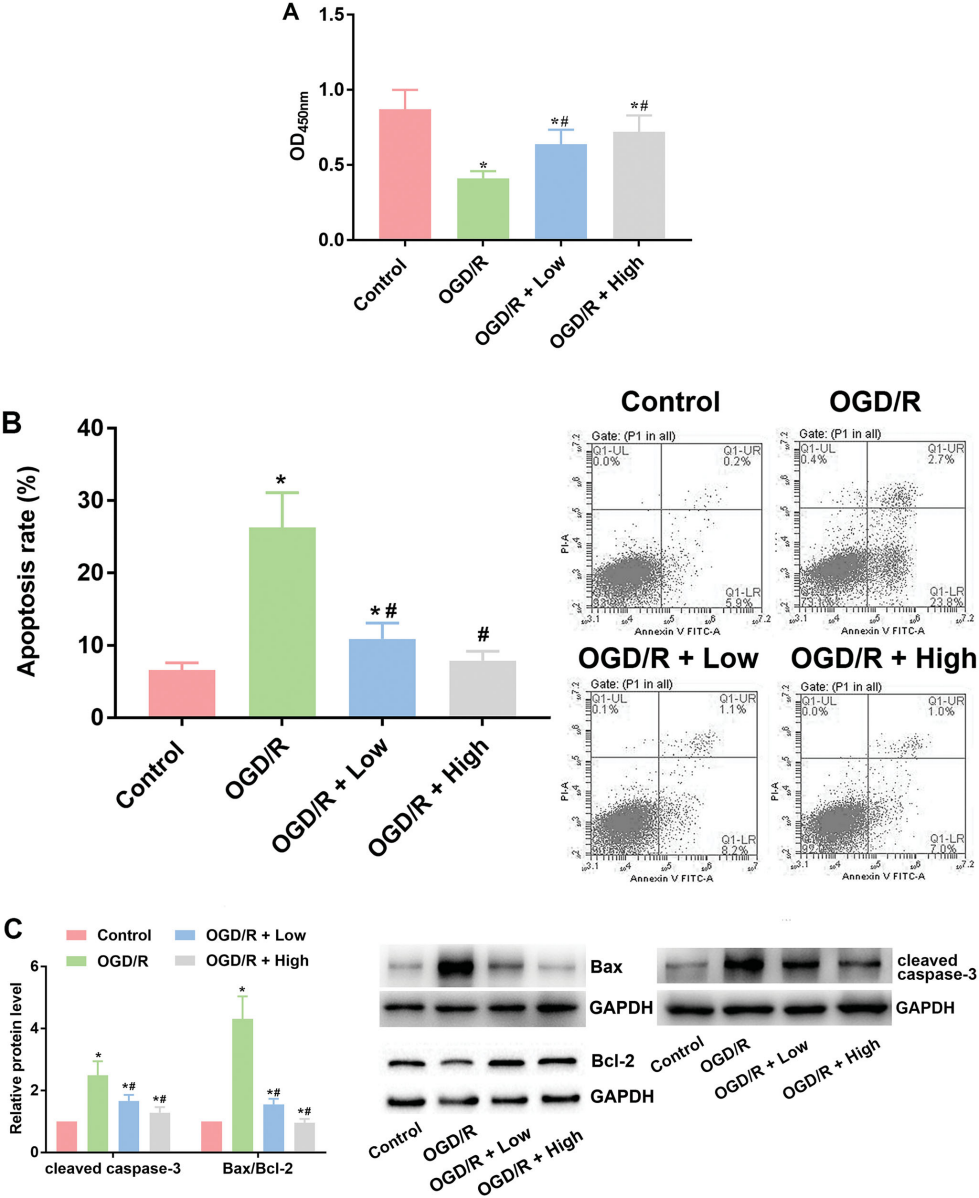
Berberine increased viability while suppressed apoptosis in OGD/R-treated cardiomyocytes. Cells were pre-treated with berberine of 10 or 20 μM 24 h before OGD/R treatment. The cell viability was detected using CCK-8 assay (A). The cell apoptosis was detected using apoptosis detection kit in a flow cytometer (B). The expression levels of apoptosis-related factors were detected using western blotting assays (C). *p< 0.05 vs. control group. ^#^*p*< 0.05 vs. OGD/R group.

The viability of cardiomyocytes was determined with a CCK-8 assay. As shown in Figure 2(A), the subjection of OGD/R administration suppressed the viability of cardiomyocytes, which was represented by the decreased OD_450_ value in OGD/R group (0.41 ± 0.05) in comparison to control group (0.87 ± 0.13) (*p* < 0.05). The impairment in cell viability was then attenuated by berberine: the treatment increased the OD_450_ values (0.64 ± 0.01 for OGD + low and 0.72 ± 0.01 for OGD/R + high) compared with the OGD/R group (*p*< 0.05) (Figure 2). Moreover, the effects of berberine showed a concentration-dependent pattern in improving cell viability: the OD_450_ value of the OGD/R + high group was significantly higher than that of the OGD/R + low group (*p*< 0.05) (Figure 2(A)).

Based on the flow cytometry results, exposure to OGD/R induced apoptosis in cardiomyocytes (26.3 ± 4.8) in comparison to control group (6.6 ± 1.0) (*p*< 0.05) (Figure 2(B)). Incubation with berberine suppressed the apoptotic rate (10.9 ± 2.2 for OGD/R + low and 7.9 ± 1.3 for OGD/R + high) in cardiomyocytes (Figure 2(B)), and the effect was exerted in a concentration-dependent pattern, with 10 μM berberine showing a stronger alleviative effect (Figure 2(B)). The results of flow cytometry were verified with western blotting assays: the level of Bcl-2 was suppressed by OGD/R administration, while the level of Bax and cleaved caspase-3 was induced (Figure 2(C)). The expression levels of the three apoptosis-related indicators were then reversed by berberine at two concentrations, confirming the antiapoptotic effects of berberine on cardiomyocytes.

### Berberine inhibited inflammatory and oxidative stress responses in OGD/R-treated cardiomyocytes

**Figure 3.**
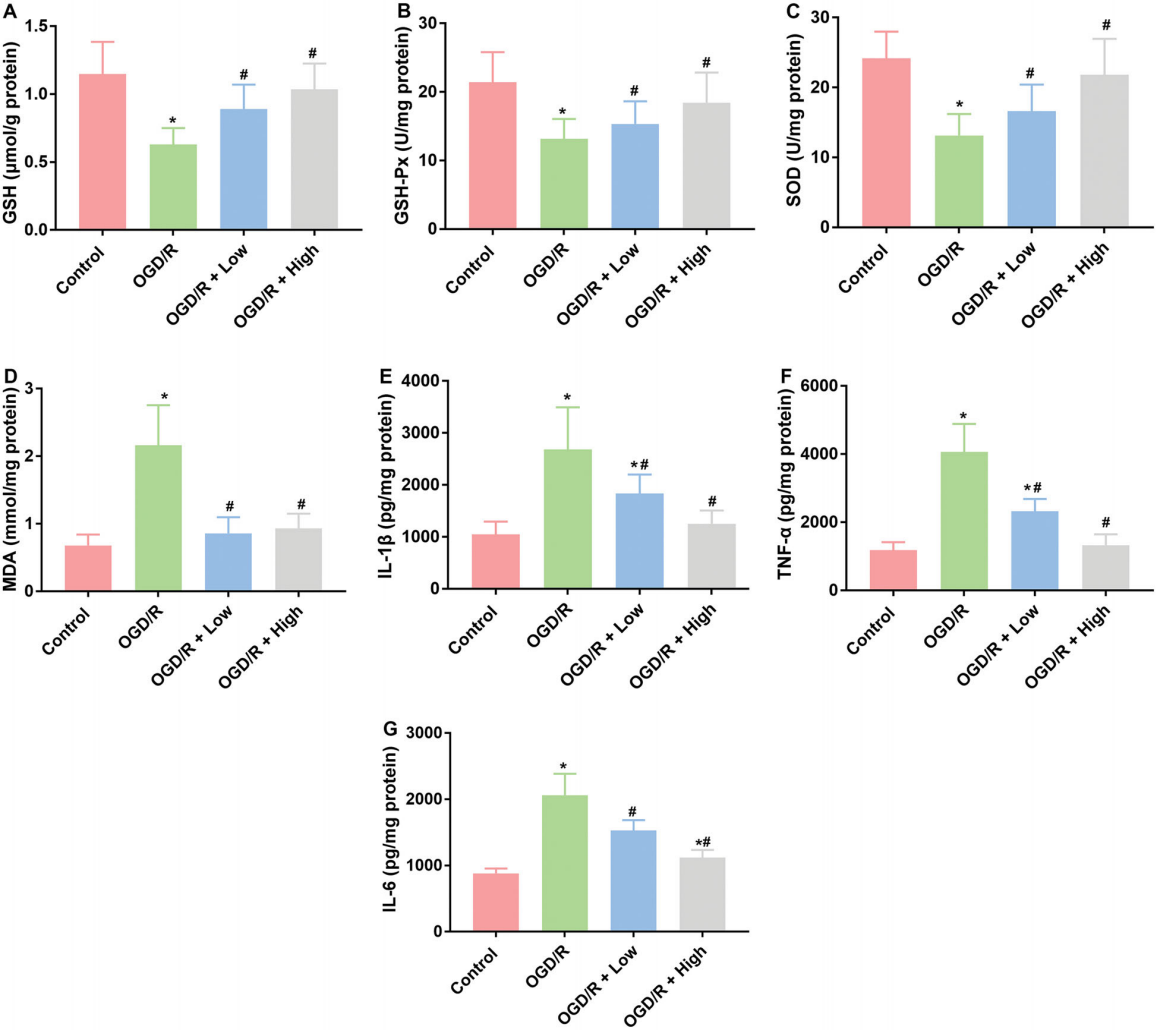
Berberine inhibited inflammation and oxidative in OGD/R-treated cardiomyocytes. Cells were pre-treated with berberine of 10 or 20 μM 24 h before OGD/R treatment. The levels of GSH (A), GSH-Px (B), SOD (C), MDA (D), IL-1β (E), TNF-α (F) and IL-6 (G) were detected using corresponding kits. *p< 0.05 vs. control group. ^#^*p*< 0.05 vs. OGD/R group.

Oxidative stress and inflammatory responses are typical characteristics associated with I/R injuries. Therefore, the effects of berberine on the production of indicators involved in these two processes were measured. OGD/R treatment suppressed the production of GSH, GSH-Px, and SOD, while induced the production of MDA, IL-1β, TNF-α, and IL-6 (Figure 3), which was indicative of the initiation of oxidative stress and the inflammatory response associated with I/R injuries. After the administration of berberine, the levels of these indicators were all restored, representing the anti-inflammatory and antioxidative stress effects of berberine (Figure 3).

### Berberine increased miR-26b-5p levels and inhibited the PTGS2/MAPK pathway in OGD/R-treated cardiomyocytes

**Figure 4.**
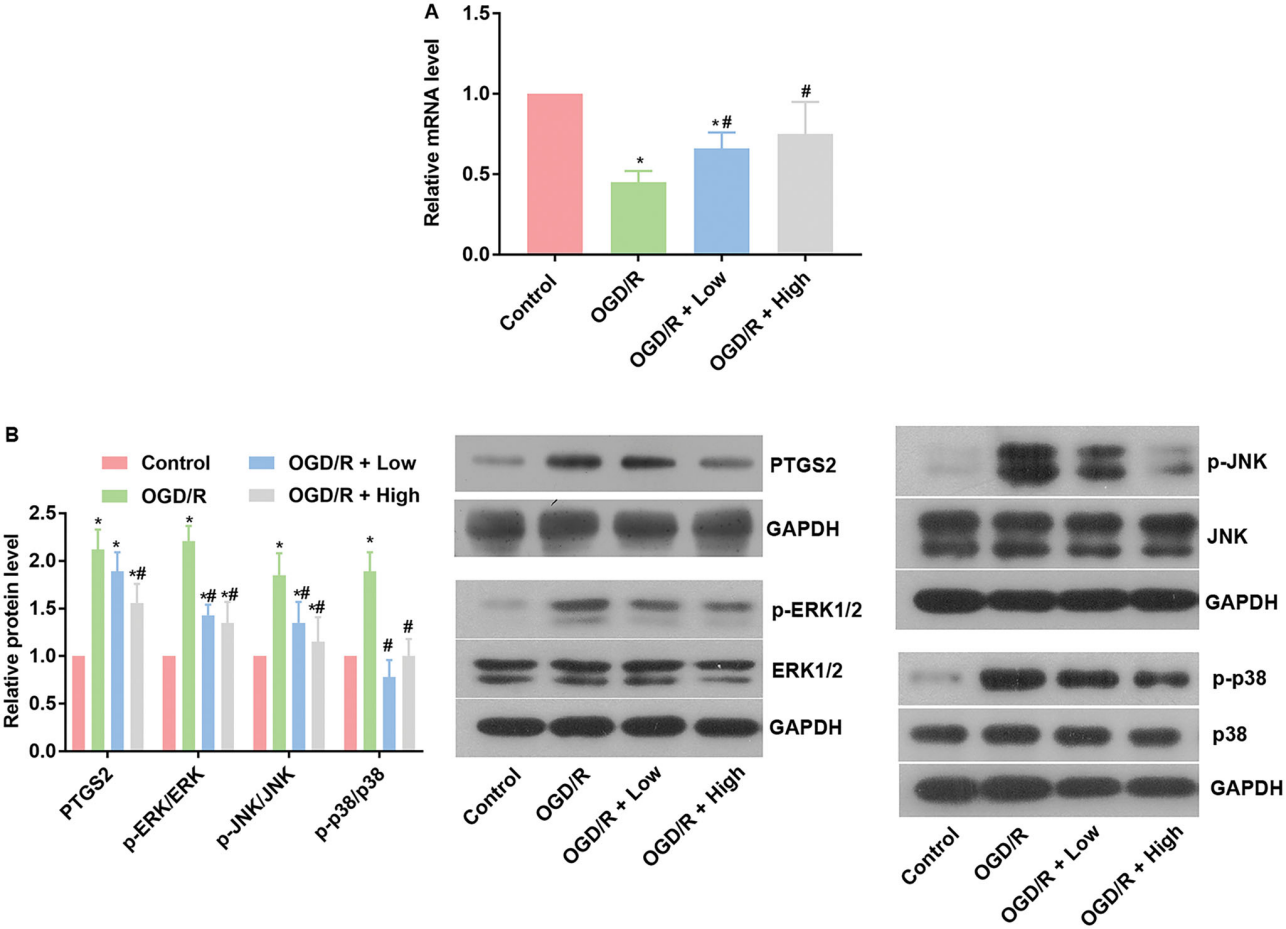
Berberine suppressed oxidative stress and inflammatory response, induced miR-26b-5p level, while inhibited the PTGS2/ERK/JNK/P38 pathway in OGD/P-treated cardiomyocytes cells. Cells were pre-treated with berberine of 10 or 20 μM 24 h before OGD/R treatment. The expression level of miR-26b-5p was detected using RT-qPCR (A). The expression levels of members in the PTGS2/ERK/JNK/P38 pathway were detected using western blotting assays (B). *p< 0.05 vs. control group. ^#^*p*< 0.05 vs. OGD/R group.

The potential interaction between berberine and miRs while protecting against OGD/R injury was explored by focussing on the activity of the miR-26b-5p/PTGS2/MAPK pathway. OGD/R administration inhibited the expression of miR-26b-5p (Figure 4(A)), which increased the levels of PTGS2, p-ERK, p-JNK, and p-p38 in comparison to the control group (*p*< 0.05) (Figure 4(B)). In cardiomyocytes treated with berberine and OGD/R, the level of miR-26b-5p was restored, and the activity of the PTGS2/MAPK pathway was suppressed (Figure 4(B)), indicating that the protective effect of berberine was related to changes in the miR-26b-5p/PTGS2/MAPK pathway axis.

### Protective effect of berberine on cardiomyocytes against OGD/R injuries depended on the induced level of miR-26b-5p

**Figure 5.**
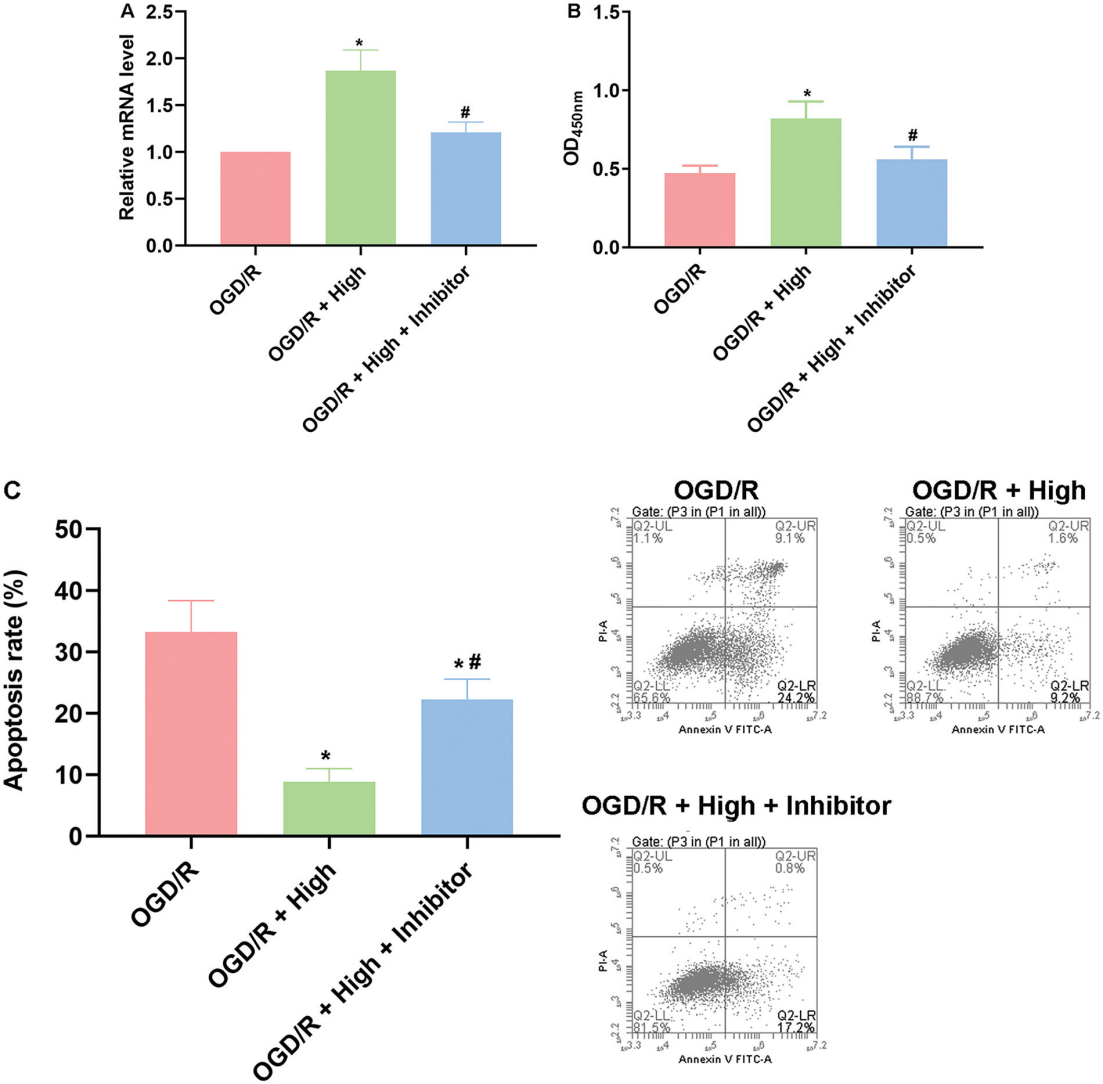
Inhibition of miR-26b-5p impaired cell viability and induced apoptosis in OGD/R and berberine-treated cardiomyocytes cells. Cells were transfected with miR-26b-5p inhibitor and then subjected to berberine and OGD/R treatments. The expression level of miR-26b-5p was detected using RT-qPCR (A). The cell viability was detected using CCK-8 assay and represented by OD value at 450 nm (B). The cell apoptosis was detected using apoptosis detection kit in a flow cytometer (C). *p< 0.05 vs. OGD/R group. ^#^*p*< 0.05 vs. OGD/R + high group.

To further explore the role of miR-26b-5p in the anti-OGD/R effect of berberine, cardiomyocytes were pretransfected with miR-26b-5p inhibitor (Figure 5(A)) and then subjected to berberine and OGD/R treatment. The results showed that with the inhibition of the constitutive level of miR-26b-5p in cardiomyocytes, the effect of berberine was impaired. The viability and apoptosis of cardiomyocytes in the OGD/R + berberine + inhibitor group were comparable to those in the OGD/R group (Figure 5(B,C)). These data collectively suggested that the induced level of miR-26-5p was indispensable for the protective effects of berberine on cardiomyocytes.

### Administration of berberine ameliorated cardiac function and structure in an I/R-injured rat model by inducing the level of miR-26b-5p

**Figure 6.**
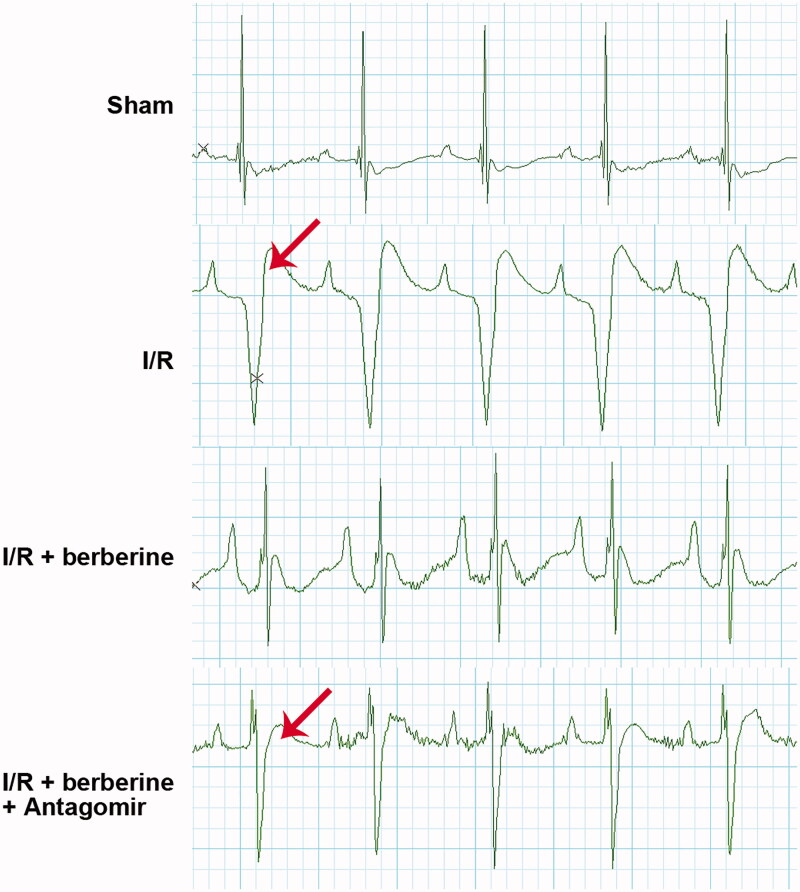
Berberine improved ECG pattern in I/R rats by inducing the level of miR-26b-5p. Arrow indicates the elevated ST segment of ECG.

**Figure 7.**
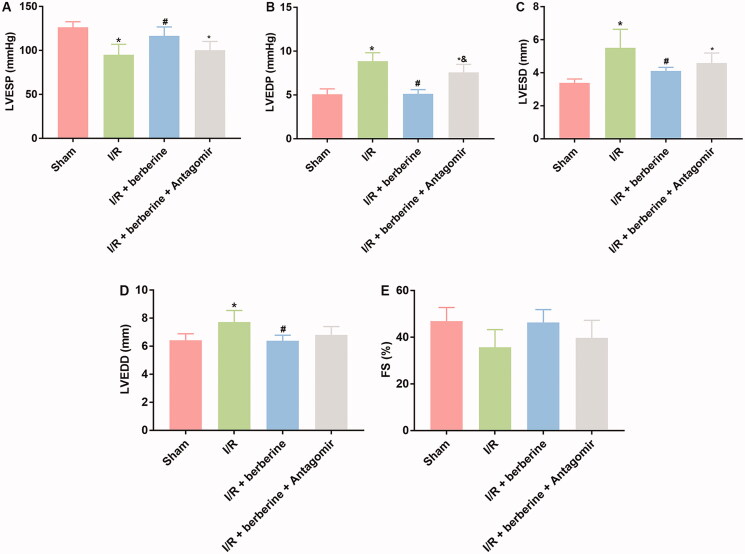
Inhibition of miR-26b-5p influenced cardiac function in model rats treated with berberine. Rats were subjected to injection of miR-26b-5p antagomir, gavaged of 50 mg/kg body weight berberine or I/R surgery in different combinations. The levels of LVESP (A), LVEDP (B), LVESD (C), LVEDD (D) and FS (E) were measured using non-invasive blood pressure system or using Philips iE33 system. *p< 0.05 vs. sham group. ^#^*p*< 0.05 vs. I/R group. ^&^*p*< 0.05 vs. I/R + berberine group.

**Figure 8.**
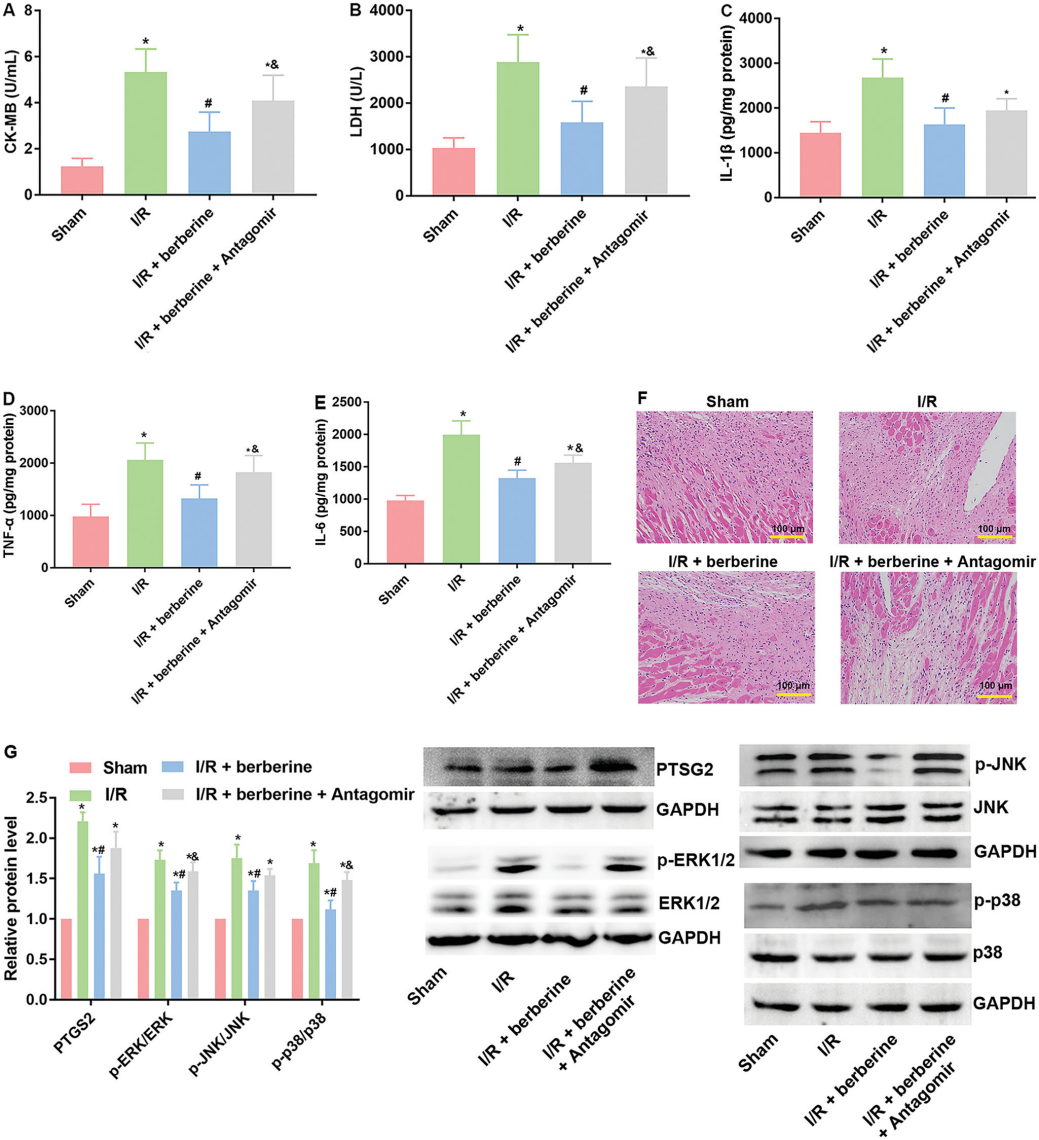
Inhibition of miR-26b-5p re-induced inflammatory response, attenuated histological destruction and suppressed PTGS2/ERK/JNK/P38 pathway in myocardial tissues in model rats treated with berberine. Rats were subjected to injection of miR-26b-5p antagomir, gavaged of 50 mg/kg body weight berberine or I/R surgery in different combinations. The myocardial levels of lactate LDH (A), CK (B), IL-1β (C), TNF-α (D) and IL-6 (E) were measured using corresponding kits. Histological changes in myocardial tissues were detected with H&E staining (F). The expression levels of members in PTGS2/ERK/JNK/P38 pathway were detected using western blotting assays (G). *p< 0.05 vs. Sham group. ^#^*p*< 0.05 vs. I/R group. ^&^*p*< 0.05 vs. I/R + berberine group.

The results of *in vitro* assays were verified with *in vivo* models. The establishment of the I/R model was first verified with ECG detection: rats in the sham group showed normal ECG. Compared with that of the control group, the ECG of the I/R group showed obvious ST segment elevation, which was reversed by berberine (Figure 6). After the inhibition of miR-26b-5p, the ECG pattern showed an increase in the ST segment again (Figure 6), suggesting that berberine could prevent I/R-induced abnormal changes in the ECG by inhibiting miR-26b-5p level. Moreover, as shown in Figure 7, I/R model establishment impaired the function of rat hearts. Berberine significantly reversed the abnormal levels of all hemodynamic parameters, including LVESP, LVEDP, LVESD, LVEDD and FS, which were impaired by model induction, representing the protective effects of berberine against I/R injury. The serum levels of LDH and CK in the I/R group were higher than those in the sham group, which was reversed by pre-treatment with berberine (Figure 8(A,B)), and the differences between the berberine groups and I/R group were statistically significant (*p*< 0.05). Moreover, the inflammatory responses associated with I/R surgery were all inhibited by berberine (Figure 8(C–E)). However, the abovementioned protective effects associated with berberine administration were all counteracted in the I/R + berberine + antagomir group: the levels of hemodynamic parameters, myocardial function-related indicators and infarction area in this group were all close to those in the I/R group (Figure 8(A–E)). Following H&E staining, the nuclei in myocardial tissue were stained blue, and the cytoplasm was stained red. The colour of collagen fibres varied from light to dark pink (Figure 8(F)). A comparison between the sham and I/R groups revealed severe injuries due to I/R. Administration of berberine before LAD ligation relieved the damage, with most myocardial cells retaining their normal structure, which was dramatically impaired by the miR-26b-5p antagomir (Figure 8(F)). Unfortunately, our data showed that even the sham surgery might induce the number of infiltrative cells: the number of infiltrative cells was higher than that of cardiomyocytes in sham group (Figure 8(F)), reminding us to be careful with the surgery in the future studies. At the molecular level, the expression levels of PTGS2, p-ERK, p-JNK and p-38 were induced by I/R surgery and suppressed by berberine (Figure 8(G)). However, with the inhibition of miR-26b-5p, the effect of berberine on the activity of the PTGS2/MAPK pathway was impaired (Figure 8(G)). Collectively, the protective effects of berberine on myocardial tissues depended on the induced level of miR-26b-5p, which was consistent with the conclusion derived from *in vitro* assays.

## Discussion

The current study affirmed that the administration of berberine could attenuate myocardial injury induced by I/R. The protective effects of the compound on myocardial tissues were exerted by inducing the expression of miR-26b-5p, which subsequently inhibited its downstream pro-IRI effectors, such as PTGS2 and MAPK members. The changing pattern of the miR-26b-5p/PTGS2/MAPK axis contributed to the suppression of inflammatory and oxidative stress in myocardial tissues and finally led to the restored function and structure of the heart. The study provided complementary information to explain the mechanism underlying the cardioprotective effects of berberine and partially revealed the interaction between miR and berberine in attenuating IRI.

Increasing evidence shows that miRs are key factors involved in the regulation of cardiovascular functions (Thum 2012; Zampetaki and Mayr 2012). Based on these theories, therapy targets regulating the level of miRs have been widely assessed for their potential to improve heart condition (Peters et al. 2020). In the current study, we selected miR-26b-5p as the therapeutic target of berberine for treating myocardial I/R injures. The protective potential of miR-26b-5p against myocardial I/R injures has been previously proven. For instance, miR-26b-5p could relieve the inflammatory response associated with myocardial infarction by modulating the PTGS2/MAPK pathway (Ge et al. 2019). Similar results were also derived in the current study. The upregulation of miR-26b-5p was associated with the inhibition of the PTGS2/MAPK pathway and suppressed the levels of inflammatory and oxidative stress responses both *in vitro* and *in vivo*. Unfortunately, our data also showed that even the sham surgery might induce the number of infiltrative cells: the number of infiltrative cells was higher than that of cardiomyocytes in sham group, reminding us of the careful surgery in the future studies. The inhibitory effect of miR-26b-5p on the MAPK pathway was exerted by its direct binding to PTGS2 (http://www.targetscan.org/cgi-bin/targetscan/vert_71/view_gene.cgi?rs=ENST00000367468.5&taxid=9606&members=miR-26-5p&showcnc=0&shownc=0&subset=1), which then contributed to the downstream inhibition of proinflammatory signal transduction, such as NF-κB (Chung et al. 2012; Himaya et al. 2012). Therefore, the development of therapies by increasing the expression of miR-26b-5p might be promising in the clinical handling of I/R injures in different organs or tissues.

In the current study, we employed berberine as an agent to increase the expression of miR-26b-5p. The protective effects of the compound on myocardial tissues against different types of injuries have been universally verified (Chen et al. 2014; Huang et al. 2015). However, few previous studies have attempted to explain the mechanism driving the anti-I/R function of berberine by exploring its interaction with miRs. Our results showed that the induced expression of miR-26b-5p was associated with the improved condition of cardiomyocytes both *in vitro* and *in vivo*, indicating the involvement of miR in the function of berberine. To confirm the indispensable role of miR-26b-5p in the anti-IRI effects of berberine on myocardial tissues, we inhibited the expression of miR in both cell and animal models. The data derived from the system with miR-26b-5p inhibition clearly showed that the effect of berberine was substantially influenced when the constitutive expression level of miR-26b-5p was low. The data to some extent showed that the protective effects of berberine on myocardial tissue depended on the increased expression of miR-26b-5p. Moreover, miR-26b-5p is a well-characterized anti-inflammatory factor in different systems (Ge et al. 2019; Ying et al. 2020), and the functions of berberine largely depend on its antioxidative and anti-inflammatory effects (Kumar et al. 2015). Based on these previous studies and our data, it was reasonable to infer that miR-26b-5p was involved in the anti-I/R function of berberine in myocardial tissues by enhancing the antioxidative and anti-inflammatory features of berberine. The conclusion was a supplement to the mechanism underlying the myocardial protective effects of berberine.

Even our work study provided some valuable information for explaining the function of berberine, our conclusion was preliminary because there are other miRs involved in the function of berberine. Thus, the interaction between miRs and berberine in attenuating I/R injures in myocardial tissues or other tissues is expected to be more complicated than that reported in the current study. The potential of miRs to promote the medicinal application of natural compounds still requires more comprehensive studies in the future.

## Conclusions

Collectively, the current study summarized the interaction between miR-26b-5p and berberine in the anti-IRI effects on myocardial tissues of the compound. The upregulated expression of miR-26b-5p by berberine contributed to the restored viability of cardiomyocytes both *in vitro* and *in vivo* via the inhibition of the PTGS2/MAPK pathway. Although our exploration is preliminary, it still provides valuable information for studies exploring the mechanism driving the diverse functions of berberine and will promote the application of compound in the future.

## Data Availability

The data will be provided by the corresponding author on request.
